# Advanced hemodialysis equipment for more eco-friendly dialysis

**DOI:** 10.1007/s11255-021-02981-w

**Published:** 2021-09-04

**Authors:** Adelheid Gauly, Nicole Fleck, Fatih Kircelli

**Affiliations:** 1 Fresenius Medical Care Deutschland GmbH, Global Medical Office, Else-Kröner-Strasse 3, 61352 Bad Homburg, Germany; 2 Fresenius Medical Care, Application Training and Clinical Support, Bad Homburg, Germany

**Keywords:** Dialysis, Waste reduction, Water consumption, Carbon footprint, Environmental sustainability

## Abstract

Healthcare in general and dialysis care in particular are contributing to resource consumption and, thus, have a notable environmental footprint. Dialysis is a life-saving therapy but it entails the use of a broad range of consumables generating waste, and consumption of water and energy for the dialysis process. Various stakeholders in the healthcare sector are called upon to develop and to take measures to save resources and to make healthcare and dialysis more sustainable. Among these stakeholders are manufacturers of dialysis equipment and water purification systems. Dialysis equipment and consumables, together with care processes need to be advanced to reduce waste generation, enhance recyclability, optimize water purification efficiency and water use. Joint efforts should thus pave the way to enable delivering green dialysis and to contribute to environmentally sustainable health care.

## Introduction

Health care is an essential pillar in a humanitarian society and should be accessible to everyone. At the same time its resource consumption, which includes water, energy and raw materials have a significant environmental impact that may have as well negative impacts on human health, an often neglected aspect [1]. The overall consumption of resources from the earth on a global level exceeds already by today the earth’s biocapacity by a factor of 1.6, as illustrated in the ‘Earth Overshoot Day’, which was August 22 in the year 2020 [2]. It is of growing importance to reduce resource consumption to postpone the Earth Overshoot Day to improve environmental sustainability in general, and in health care [2].

Energy and material consumption ultimately cause emissions of greenhouse gas including CO_2_. The United Nations have stipulated the Paris Agreement to limit global warming, this to be achieved amongst others by reducing significantly greenhouse gas emissions [3]. Between 3 and over 10% of a country's CO_2_ emissions originate from the health care sector, with a per capita carbon footprint in 2014 of in average 0.6 tCO_2_, ranging from 0.06 tCO_2_ in India to 1.51 tCO_2_ in the US [4].

The dialysis sector is one of the resource consuming fields with higher than healthcare average values of carbon footprint. A wide spread of values has been calculated for hemodialysis (HD) e.g. 3.8 tCO_2_ equivalents (Eq) per patient and year in a UK centre [5], 10.2 tCO_2_ Eq calculated for an Australian centre [6], and for peritoneal dialysis (PD) 1.4 tCO_2_ Eq per patient and year, as estimated in a Chinese study [7]. Differences may in part be attributable to different assumptions, parameters that are included in the calculation, type of energy sources, etc.

The HD treatment itself contributes with a broad range of components to this resource consumption. This includes consumables used for each treatment such as dialyzer, bloodlines, needles, syringes, concentrates and medications. In addition, non-treatment factors such as patient travel and transportation of goods, energy and water for water purification systems to produce dialysis fluid, and energy to heat the dialysis fluid and to operate the dialysis machine and dialysis centre add up to the dialysis associated resource consumption.

This is summing up to a remarkable quantity, considering the current number of approx. 3 Mio patients worldwide being chronically treated with HD [8]. The number of patients on kidney replacement therapies continues to rise due to the increasing proportion of people at risk. In developed countries e.g. an ageing society or increasing prevalence of diabetes results in an increasing incidence of chronic kidney disease. In addition, more patients in developing countries are being offered kidney replacement therapy [9], thus the overall environmental impact will continue to increase.

Strategies are required to balance the goals to reduce resource consumption and greenhouse gas emission, and to ensure uncompromised patient care. Various initiatives have been launched to address environmental sustainability in health care in general and specifically in kidney care [10], such as the Green Dialysis Initiative [11], or the ERA-EDTA initiative for greener healthcare [12]. Efforts to provide environmentally friendly and sustainable health and dialysis services are certainly diverse, and include infrastructural and procedural measures in the centre organization and treatment delivery to reduce resource consumption [13, 14]. The need for measures to effectively address the environmental impact of dialysis management has been comprehensively formulated in a position statement by the Italian Society of Nephrology [15].

To move towards the goal of 'green dialysis' [16], also manufacturers of dialysis equipment are demanded to contribute. They are encouraged to include environmental sustainability as a component of a holistic approach to design and develop advanced dialysis equipment that meets a wide range of current requirements.

In this review we specifically illustrate these requirements for dialysis care, discuss steps and provide examples on how advanced dialysis equipment and dialysis care could contribute to more environmentally sustainable health care, focusing on Fresenius Medical Care initiatives and equipment.

## Waste reduction through advanced dialysis systems

Each HD session generates several kilograms of waste, the larger part made of plastic. Depending on local waste management systems and regulations, a significant proportion of this waste is classified as hazardous since it has been in contact with blood, and implies special handling and disposal, which is usually more expensive and laborious than that of non-hazardous waste. A thorough quantitative analysis of dialysis-associated waste based on four different HD systems resulted in between 1.5 and 8 kg of waste per treatment depending on the HD system and the emptying policies of the extracorporeal systems after treatment [17]. Other sources have assessed an average weight of solid waste generated per patient and treatment of 2.5 kg [18, 19], without considering outer cardboard packaging material. Assuming this number as an average, a mean number of 156 HD treatments per patient and year and a current worldwide number of 3,160,000 HD patients in 2019 [8], this corresponds to an estimated yearly global waste production of approximately (approx.) 1.2 Million (Mio.) t, of which a significant proportion may be classified as hazardous. Parallel to the forecasted increase by approx. 6% in patients entering an HD programme [8], the amount of waste will increase accordingly, if no appropriate measures of waste reduction are put in place.

Among the strategies to reduce, recycle, and reuse waste, the most efficient way would be to reduce, i.e. generate less waste from the outset. This has been recognized and triggered initiatives at dialysis centre level mainly based on local and national regulations [18], but also manufacturers are called upon taking up this challenge.

This message was taken by many manufacturers to design systems and disposables that generate less waste. A new HD system, the 6008 CAREsystem (Fresenius Medical Care, Bad Homburg, Germany) has been developed, where the conventional blood line system, and in case of on-line hemodiafiltration additionally a substitution line is replaced by an all-in-one cassette system unifying all the components of the extracorporeal circuit. This not only reduces the total disposable weight but also targets simplification of operation of the HD system. Through the design of the cassette and use of lighter material (polyolefines), the weight of the unused disposable is approx. 100 g less than that of blood lines used for the 5008 CorDiax HD system (Fresenius Medical Care, Bad Homburg, Germany) and the Gambro Artis system (Gambro, Lund, Sweden). After treatment, the measured weight is 298 g for the cassette system used with the 6008 system, 487 g for bloodlines and disposables used with the 5008 system, and 514 g for those used with the Gambro Artis system, respectively, all excluding the dialyzer [20]. Another similar investigation measured 150 g less waste per treatment with the 6008 compared to the 5008 HD system [21]. For a typical centre performing around 10,000 treatments per year, this leads to a potential of clinical (hazardous) waste reduction by approx. 1500–2000 kg and accordingly reduced cost for waste disposal, which varies according to the local waste management systems [20]. These positive effects would multiply with increasing patient numbers.

Another option in the HD treatment to reduce waste by design is performing on-line priming and rinsing in the set-up phase of the treatment, on-line infusions as necessary during treatments and on-line reinfusion at the end of the session both in HD and on-line HDF instead of applying saline from an extra bag [22, 23]. This change in practice may save both waste and cost from the omitted saline bags [18]. Another approach to reduce waste are integrated priming fluid drainage devices avoiding extra drainage bags as in the Artis Physio HD system (Baxter Healthcare Corporation, Deerfield, IL, USA) [24].

Further, the concentrate supply can be addressed to optimize resource management. Each treatment requires dialysis fluid which is either delivered through central concentrate supply or is prepared at bedside from concentrates provided in a plastic canister and/or dry powder and water. An alternative to such canisters are flexible bags providing the acid concentrate, which require less packaging material and have therefore less weight than a canister. An example is the so-called Smartbag (Fresenius Medical Care, Bad Homburg, Germany) [25], that can be folded after treatment and easily emptied, thus not only the weight of the waste, but also the volume can be reduced. A similar approach for basic concentrates has long been used (i.e. Bibag, Fresenius Medical Care, Bad Homburg, Germany).

In addition to the absolute weight reduction, the waste composition is also relevant to ensure safe management of healthcare waste. Clinical waste should be separated into infectious and non-infectious waste to separate for incineration or landfill and recycling, respectively [26]. For those components, which are incinerated—this includes often the extracorporeal system in HD, it would be desirable that, as for many medical consumables, the use of polyvinyl chloride (PVC) is replaced by chlorine-free polymers in order to minimize during combustion the formation of dioxins and furans, which are generated at insufficiently high temperatures [26]. Moreover, the use of medical devices containing PVC should be limited over the patient’s time on dialysis due to health risks associated with plasticizers integrated in PVC polymers, that potentially migrate depending on the type of plasticizer [19, 27]. Although technologies are emerging to recycle PVC [28], extracorporeal blood circuits are rather prone to be incinerated as clinical waste with previous blood contact. In the 6008 cassette system many components, such as the cassette itself, are made of polyolefins, chlorine-free polymers as a replacement for PVC. This polymer, branded as Biofine^®^ (Fresenius Medical Care, Bad Homburg, Germany) may further reduce patient exposure to PVC and plasticizers [29] as well as the environmental burden of health care waste.

Consideration of the aforementioned aspects and additional points is converging in a so-called Life Cycle Analysis on products [30]. This covers different impact categories, including climate change and resource depletion. The product life cycle stages can follow the cradle-to-grave principle and range from raw materials, manufacturing, packaging, transportation/distribution, use, and waste management (End-of-Life). Such analysis on packaging of dialysis concentrates to produce approx. 210 L dialysis fluid revealed that compared to a 6 L, 1 + 34 canister an improvement in 15 environmental impact categories on average by 6% could be achieved with a 4.7 L, 1 + 44 canister, and by further 3% with the flexible Smartbag (4.7 L, 1 + 44). An investigated dialyzer (FX classix 80) achieved, amongst others through use of lightweight polypropylenes, an ecological advantage in 14 out of 15 environmental impact categories resulting in an average improvement of 42% in comparison to a predecessor dialyzer (HF 80S, all Fresenius Medical Care, Bad Homburg, Germany) (Unpublished reports by Fresenius Medical Care).

Appropriate waste management holds an important place for a greener dialysis. There are various practices of handling disposables after the HD session, which have been assessed by Piccoli et al. [17]. Emptying the system and separating from non-hazardous waste after disconnecting from the patient (called 'careful-optimal') obviously generates less waste volume and weight, whereas the other extreme of not emptying the system and not separating hazardous from other waste (called 'careless max') may increase the amount of waste by a factor of up to 7 [17]. Smart dialysis systems can support practices to minimize waste after treatment. Such an example is the new 6008 CAREsystem which allows to automatically empty the extracorporeal system, thus, supporting the 'careful-optimal' practice and reducing further the weight of hazardous waste without having to do this laboriously by hand.

## Water sparing strategies through optimized dialysate flow

Hemodialysis requires purified water to prepare the dialysis fluid of appropriate quality. Water preparation includes filtration steps, ion exchange and reverse osmosis (RO). During reverse osmosis water passes, driven by hydrostatic pressure, through ion-exclusion semipermeable membranes to separate solutes (dissolved solutes and insoluble impurities) from the solvent, i.e. from water. The filtered solutes remain in the so-called reject water. The proportion of reject volume determines the efficiency of RO systems. This can be low, rejecting up to 75% of the inlet water volume, thus producing a yield as low as 25% [16, 31]. Therefore, it can be anticipated that with a dialysate flow of 500 mL/min for 4 h not only the 120 L water for dialysis fluid are required, but up to 480 L of water, taking also reject water into account. Additional water is used during the preparation and disinfection cycle adding to the water need by the actual treatment. This illustrates the dimension of water saving potential implementing more efficient RO systems in the dialysis centre. Modern systems can achieve a yield of approx. 80% of RO water [32]. A recent analysis in the French Nephrocare network demonstrated that, together with implementation of several strategies such as procedural reporting measures, replacement of the HD machinery by more modern types and the implementation of a more efficient water purification system with a lower proportion of reject water of only 20% can lead to a decrease of average water consumption per treatment of approx. 50% [14].

The reject water is often directly drained, and therefore lost. However, some units have reported creative ways of using this reject water such as for toilet flushing, plant watering, that offers water savings at other points within the system [33].

Moreover, energy savings on heating water for the dialysis fluid can be achieved with heat exchange systems using the residual heat in the drained dialysate to heat fresh water (B. Braun Avitum AG, Melsungen, Germany) [34].

Dialysis efficacy in terms of Kt/V or solute removal is an outcome mainly related, but not limited to blood flow, dialysate flow, treatment time and dialysis membrane surface area. There is a proportional effect of both, blood and dialysate flow on clearance and Kt/V, however, both reach a plateau at higher rates. Therefore several studies have investigated to which extent limiting dialysate flow affects dialysis efficacy [32]. It turned out that dialysis dose was lowered relatively less than dialysate flow. Thus, the potential savings in dialysis fluid and water consumption should not compromise dialysis adequacy. Long-term effects were addressed by a group from Colombia who was able to show that the reduction of the dialysate flow from 500 to 400 mL/min over 2 years not only had no significant influence on clinical parameters and Kt/V, but also not on mortality [35, 36].

Recent HD systems have moved from pre-defined dialysate flow rates (e.g. 300, 500, 800 mL/min) to an individual setting, or an automatic adjustment with a fixed factor of the dialysate flow to the blood flow. Both allows more flexibility of choosing the appropriate dialysate flow as an optimal adjustment to the current blood flow. If the dialysate flow follows the actually applied blood flow in a ratio of e.g. 1.2: 1, it is adjusted in this ratio also when blood flow is reduced during e.g. internal test, thus inefficient dialysate fluid wasting shall be minimized. This so-called AutoFlow option available in both the 5008 CorDiax and the 6008 CAREsystem can be adjusted between 1: 1 and 1.5: 1 depending on the clinical need for each individual patient. This option has been investigated in several studies to quantify dialysate savings versus the achieved dialysis dose. In the on-line HDF mode, the AutoFlow function decreased the consumed dialysis fluid by 8%, including also approx. 16% of the total volume used as substitution fluid, while at the same time the achieved dialysis dose in terms of Kt/V was slightly higher than with standard HD [37]. Other studies reported even higher reductions in dialysate consumption. Kult and Stapf applied AutoFlow in HD, and additionally AutoSub (substitution flow adapted to transmembrane pressure) in the on-line HDF mode, and achieved an approx. 30 and 19% lower dialysis fluid consumption [38]. A reduction of dialysate volume by approx. 20% through application of AutoFlow instead of manually fixed dialysate flow rate was reported by Alayoud et al. [39]. Analogous to the results of studies applying a generally reduced dialysate flow rate [35], the dialysis savings achieved with the AutoFlow function should with each treatment lead to greater savings in mains water and of energy for the production and heating of purified water without compromising clinical outcome.

## Reducing carbon footprint through home dialysis

In the attempt to reduce the carbon footprint of dialysis [5, 6], alternative dialysis therapies beyond in-centre HD are worth considering. During the patient's journey along various options of kidney replacement therapies, home-based treatments, either PD or home HD are gaining increased attention, since they allow the patient to be more independent, flexible and self-determined.

PD patients usually apply a cumulative fluid volume of between 8 L/day in continuous ambulatory peritoneal dialysis (CAPD) and up to 15 L/day in automated peritoneal dialysis (APD) depending on the prescription as well as the local and national reimbursement schemes, amounting to a yearly PD fluid consumption of between 3000 and 5500 L. In CAPD, electricity is required for warming of the solutions, in APD additionally to operate the PD cycler overnight. Waste produced with PD results from the solution systems including bags, tubing and drainage sets, and caps, consisting of different plastic materials. There is data on daily waste amounting to between approx. 0.6 [40, 41] to 1.69 kg/day [19], depending on modality, PD system, number and size of prescribed bags. Accordingly, PD can be advantageous compared to HD in view of produced waste and water used. However, for both modalities water, energy and raw material consumption for manufacturing needs to be taken into consideration, for PD also the carbon footprint of regular solution transport and delivery to the patient’s home, in order to balance different dialysis modalities for their overall environmental footprint.

PD patients may also require switching to HD due to different factors, mainly due to ultrafiltration failure and loss of residual renal function over time which eventually leads to dialysis inadequacy. In this case, home HD and in-centre HD are options depending on the shared decision making between the patient and the physician, availability of a home HD programme and other potential contributing factors.

By end of 2017 the percentage of dialysis patients being treated with home HD was still low, ranging from e.g. 1.8% in the US [42], over 4.5% in the UK [43], to 8 and 18% in Australia and New Zealand [44]. These figures contrast with preferences of patients, who would prefer home treatment based on provided information and involvement in modality choice [45]. Resource savings of performing home HD result mainly from the saving of patient travel to and from the dialysis center as well as a low dialysate flow used in some of these systems. The extent to which not needed patient travel reduces the carbon footprint naturally depends on the distance between the dialysis center and the patient's home. An exemplary calculation is based on an average distance of 17 km to the next dialysis center, which amounts to a saving of approx. 5300 km per year [46]. Accordingly, the calculated savings based on variable assumptions vary between 0.73 and 0.96 t CO_2_ Eq/patient*year [5, 46]. A comparison taking into consideration the resource consumption and emission relating to the delivery of three treatments of 4 h either in-centre or at home results in savings of approx. 13% CO_2_ Eq with home HD [5]. Such savings can be further enhanced by energy and waste saving equipment as outlined above or HD equipment specifically designed for home treatment. Connor et al. included the NxStage systems for home HD in their analysis, which revealed a potential for a reduced carbon footprint of the treatment by approx. 70% through, amongst others, lower energy consumption for the operation of the machine, more efficient water purification, further lower water consumption, low dialysate volume treatments and, consequently, lower shipping volume of dialysate concentrates [5, 47].

These savings in the home HD setting may be offset to some extent, if the patient performs more than three treatments per week, as one or more additional sets of disposables are used per week [5]. On the other hand, this allows adding e.g. one weekly session at home to increase cumulative treatment time and frequency, and by this means the weekly dialysis dose at a carbon footprint comparable to the in-centre three times 4 h schedule. The usage of equipment producing less waste or being specifically designed for home HD helps to limit additional waste and emissions resulting from the use of consumables, even though more detailed analyses including upcoming home dialysis systems are needed to explore the potential of home dialysis to make kidney replacement therapy more environmentally-friendly.

## Outlook and conclusion

The health care and dialysis community is undertaking many efforts to limit resource consumption and greenhouse gas emissions. To combat ongoing climate change and taking into account its health effects and the maintenance of healthcare, it is important to address in a holistic approach the entire spectrum of more environmentally friendly approaches to move towards the goal of 'Green Dialysis'.

One key step on the road to more eco-friendly dialysis is the design and use of dialysis equipment that reduces resource consumption and eventually the dialysis treatment's carbon footprint. Many manufacturers have already developed systems producing less waste and using less energy. With finite resources, product design should focus even more on the full life-cycle of a product including recyclability of certain components than it is doing now. In addition, progress towards green dialysis in terms of water treatment technologies as well as efficient management of dialysate consumption will be a key approach.

Manufacturers will certainly proceed on that path, also together with efforts to decrease water and energy consumption during the production process. This has not been addressed here in detail but will be in the interest of the manufacturers to produce dialysis systems and consumables economically and ecologically. Besides the equipment as such also initiatives to review and revise centre practices in the day-to-day usage of dialysis systems and established processes of resource and waste management need to be addressed. Such environmental management system includes enhancing awareness, implementing education and tracking programmes for all stakeholders in health care to monitor needs and achievements.

Finally, choosing the right place for the treatment, whether it is at home or in-center, and the right modality may allow improved resource savings in conjunction with a potential for enhanced patient satisfaction. If all approaches to optimally use resources are well balanced to ensure patient care without compromising treatment quality, this may be one important step to support sustainability of dialysis care. By this means, green dialysis is not only serving to save resources, but can be seen as a holistic approach to improve patient care and to support the entire community.

## Data Availability

Not applicable (review).
